# Posttransfusion Haematocrit Equilibration: Timing Posttransfusion Haematocrit Check in Neonates at the National Hospital, Abuja, Nigeria

**DOI:** 10.1155/2015/175867

**Published:** 2015-03-15

**Authors:** L. I. Audu, A. T. Otuneye, A. B. Mairami, L. J. Mshelia, V. E. Nwatah

**Affiliations:** Neonatal Unit, National Hospital Abuja, Plot 132, National Hospital Road, Central Business District, PMB 425, Abuja, Nigeria

## Abstract

Anaemia is a common morbidity in the NICU and often requires transfusion of packed red blood cells. Haematocrit equilibration following red cell transfusion occurs over time ultimately resulting in a stable packed cell volume (PCV). Knowledge of this equilibration process is pertinent in the accurate timing of posttransfusion (PT) PCV. We conducted a prospective study to determine an appropriate timing for PT PCV estimation on 47 stable anaemic babies at the Neonatal Unit of National Hospital, Abuja. Values of PCV were determined before transfusion and at 1, 6, 12, 24, and 48 hours posttransfusion. Forty of the recruited neonates and young infants were analyzed. Their gestational age range was 26 to 40 weeks. 1-hour PT PCV (48.5% ± 5.5%) was similar to the 6-hour PT PCV (47.8% ± 5.6%) *P* = 0.516, but both were significantly different from the 12-hour (46.8% ± 5.9%), 24-hour (45.9 ± 5.8%), and 48-hour (45.4% ± 6.2%) PT PCVs. The 12-hour PT PCV was similar to the 24-hour and 48-hour PT PCVs (*P* = 0.237 and 0.063, resp.). We concluded that, in stable nonhaemorrhaging and nonhaemolysing young infants, the estimated timing of haematocrit equilibration and, consequently, posttransfusion PCV is 12 hours after red blood cell transfusion.

## 1. Introduction

Anaemia is a common morbidity in the neonatal intensive care unit (NICU) and this arises predominantly from frequent iatrogenic blood draws necessitated by the need for laboratory investigations and monitoring. The high prevalence of haemolytic conditions in Nigeria has also contributed to increased neonatal transfusion rates in the country [1, 2].

There are different formulas for estimating volume of required packed cells with varying degrees of accuracy [3–5]. This may result in variability in the correlation between the volume of transfused blood and posttransfusion packed cell volume (PCV). It is therefore important to routinely estimate posttransfusion PCV.

Following red blood cell transfusion in stable patients, haematocrit equilibration occurs over time before a stable PCV level is reached. Knowledge of the duration of this equilibration process is pertinent in the accurate timing of posttransfusion PCV check which should be done at the earliest possible time to avoid needless prolongation of hospital stay as well as delay in completing packed cell replacement in those whose posttransfusion PCV may fall below the expected values. Although there is no consensus on the most appropriate timing of posttransfusion PCV, some studies have suggested early (<1 hour) posttransfusion haematocrit check. Wiesen et al. [6] in their study of adult medical inpatients reported that there was no difference in the posttransfusion PCV at 15 min and 24 hours. Similarly, Glatstein et al. [7] measured posttransfusion haematocrit in neonates at 15 min and 6 hours and obtained similar values both of which were significantly different from the pretransfusion haematocrit. They concluded that posttransfusion PCV can be done 15 minutes after completing transfusion. While trying to evaluate the correlation of transfused volume of red cells with change in haematocrit, Elzik et al. [8] took blood samples for haematocrit levels 24 hours after transfusion. The assumption, although not stated, was that posttransfusion equilibration would have occurred by 24 hours.

At the Neonatal Unit of the National Hospital, Abuja, posttransfusion PCV check is routinely carried out empirically 48 hours after completing red blood cell transfusion. In some instances, while waiting for predischarge posttransfusion PCV, babies occupy bed space, incurring additional hospital bill and denying other incoming sick babies admission/bed space.

There is no doubt that there is dearth of evidence-based information on the most appropriate timing for posttransfusion PCV estimation. The objective of this study was therefore to determine an appropriate timing for posttransfusion PCV check in stable anaemic babies at the Neonatal Unit of the National Hospital, Abuja. We hypothesized that significant differences exist in the values of posttransfusion haematocrit estimates done at 1, 6, 12, and 24 hours compared to haematocrit estimates at 48 hours. It was hoped that an evidence-based recommendation on the appropriate timing of posttransfusion PCV check would emanate from this study.

## 2. Subjects and Methods

### 2.1. Study Site

The study was carried out at the Neonatal Unit of the National Hospital located in the central area of Abuja, the Federal Capital Territory of Nigeria. This is a 300-bed tertiary referral health center affiliated to the University of Abuja. The paediatric department of the hospital has 120-bed capacity, about 30% of which is devoted to neonatal care. The Neonatal Unit of the National Hospital, Abuja, consists of 40-cot/incubator space divided into an inborn section for babies delivered within the hospital and an outborn section for babies brought/referred from other hospitals.

This was a prospective cohort study of babies who required blood transfusion in the newborn unit from January to June 2014.

The study was conducted over a period of 6 months from January 2014 to June 2014. A baby was eligible if the PCV level fell within the limit set by the unit protocol for blood transfusion (<35% in the first week of life, <30% from 8 days to 14 days, and<25% for older babies). This also takes into consideration the presence or absence of signs of cardiac decompensation or recurrent apnea. The target (expected posttransfusion) PCV was 45% for babies less than 2 weeks and 40% for older babies. Any baby who had evidence of* ongoing* blood loss or* continued* haemolysis was excluded. Capillary PCV was done twice at an interval of 24 hrs and those babies with a PCV difference of >2% (0.6 g/dL) [8] were assumed to be unstable (ongoing blood loss or haemolysis). Babies who required whole blood transfusion as well as those who died before completing the posttransfusion PCV measurements were also excluded. In our unit, furosemide is not routinely administered during red cell transfusion except for babies with clinical evidence of heart failure or fluid retention.

The ethics committee of the hospital granted ethical approval for the study while parental consent for participation in the study was obtained from the mothers before commencement of study.

For every eligible patient, relevant sociodemographic maternal and neonatal data were documented. The cause of anaemia (if known) was also noted and documented. The volume of packed cells to be transfused was calculated as *V* (mL) = 3 × wt (kg) × (expected change in haemoglobin.) [4]. This volume was given as a continuous infusion using an infusion pump or given in aliquots over a period of 3-4 hours [9]. Time of commencement and completion of transfusion was documented. Capillary blood samples were then obtained at 1 hour, 6 hours, 12 hours, 24 hours, and 48 hours after completion of transfusion by our residents for determination of PCV using Biofuge.Haemo Centrifuge (Heraeus Instruments) at 10,000 revolutions per minute. Values of PCV were read using haematocrit reader.

### 2.2. Statistical Analysis

Data generated were analyzed using SPSS version 17. Mean (SD) values were computed for pre- and posttransfusion PCV at the specified periods and, using the Student *t*-test, levels of statistical significance (*P* values) were generated for the differences in the mean values of PCV. *P* values less than 0.05 were considered significant. Levels of significance were also computed for the difference between the target posttransfusion PCV and the mean PCV observed at the specified times.

## 3. Results

Forty-seven infants who met the inclusion criteria were prospectively recruited into the study. Seven of them were excluded from analysis because of incomplete data (missing posttransfusion PCV results). The remaining 40 infants formed the subject of this analysis. These consisted of neonates and young infants aged 1 to 91 days (22.7 ± 20.6), with a gestational age range of 26 weeks to 40 weeks. Five babies (12.5%) were VLBW and 10 (25.0%) were ELBW. Twenty-six babies (65%) had blood transfusion within the neonatal period while most of the babies with anaemia of prematurity were transfused beyond the 28-day postnatal age. Eighteen babies (45%) had anaemia of prematurity while 7 had haemolytic anaemia associated with jaundice (ABO isoimmunization = 6, sepsis = 1).  Table 1 shows the demographic characteristics of the subjects and the aetiology of anaemia.

The pretransfusion PCV was 32.1% ± 6.2% and this was significantly lower than the 1-hour posttransfusion PCV (48.5% ± 5.5%, *P* = 0.0000). As shown in Table 2, 1-hour posttransfusion PCV was similar to the 6-hour posttransfusion PCV (47.8% ± 5.6%) *P* = 0.516, but both were significantly different from the 12-hour, 24-hour, and 48-hour posttransfusion PCVs. The 12-hour posttransfusion PCV was similar to the 24-hour and 48-hour posttransfusion PCVs (*P* = 0.237 and 0.063, resp.). The difference between the 1-hour posttransfusion PCV and the 12–48-hour PCV varied from 1.3% to 3.5% (0.4 g/dL to 0.9 g/dL Hb).

The mean 1-hour and 6-hour posttransfusion PCVs were significantly different from the target posttransfusion PCV (*P* = 0.048 and 0.046, resp.). However the 12-hour, 24-hour, and 48-hour posttransfusion PCVs were similar to the posttransfusion target PCV (*P* = 0.157, 0.495, and 0.683, resp.). This is shown in Table 3.

**Figure 1:**
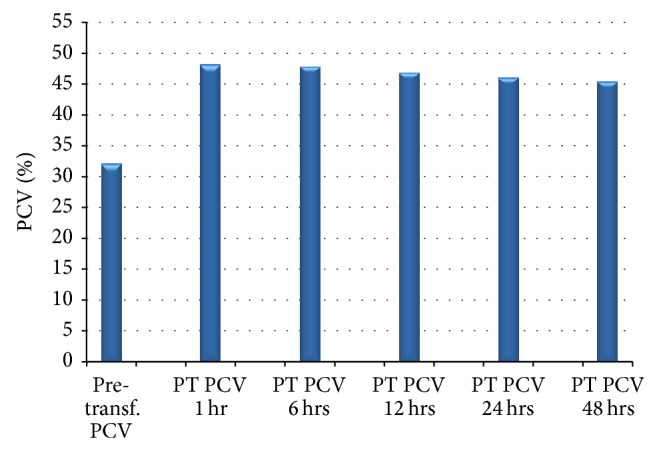
Mean PCV values before and after packed cell blood transfusion.

Figure 1 is an illustration of the changes in PCV following packed red cell transfusion. The immediate rapid increase (1–6 hours) was followed by a gradual drop in 6–12 hours and maintenance of a steady state between 12 and 48 hours.

## 4. Discussion

Our study shows the expected immediate rise in PCV posttransfusion agreeing with results of previous studies. Although this significant difference between the pre- and posttransfusion PCV was sustained throughout the period of observation (48 hours), the change in PCV posttransfusion followed a unique pattern which suggests that the equilibration process that probably began while red blood cell transfusion was ongoing terminated at about 12-hour posttransfusion beyond which no significant difference in PCV was noted. Transfused packed red blood cells contain extracellular electrolytes (sodium, potassium, chloride, and bicarbonates) which contribute some solute load ≈ 34 mmol/L to the extracellular space, resulting in the movement of water from the intracellular compartment into the extracellular compartment. This may account for the significant drop in PCV values observed between 6 hours and 12 hours after transfusion in our study. We chose stable babies for this study to ensure elimination of the confounding effect of ongoing blood depletion (haemorrhage/haemolysis) on the equilibration process. The results from our study would suggest that PCV check could be done 12 hours after completing red blood cell transfusion in neonates.

We considered the effect of repeated posttransfusion blood draws on the declining PCV from 1 hour to 48 hours. The average blood volume per capillary draw is 0.07 mL and 0.35 mLs for 5 samples. The mean weight of babies in the study group was 3.3 kg. Given that 6 mLs/Kg of whole blood transfused to a baby increases the haematocrit by about 3%, 0.35 mLs/3.3 Kg of blood drawn from baby for PCVs will expectedly produce a deficit of 0.05% in PCV from 1-hour posttransfusion to 48-hour posttransfusion. In our study the difference in PCV from 1 to 48 hours after transfusion was 2.7%, which exceeded the deficit estimated from the blood draws.

While a coefficient of variation of about 7% has been reported for capillary PCV [10], some of the precautions we took while taking the samples (avoiding squeezing out blood from prick site and ensuring babies were in supine position) would necessarily limit the changes in body fluid distribution that may partly account for this variability.

Although Elzik et al. [8] demonstrated that PCV at 15 minutes and 6 hours after red cell transfusion in stable neonates were similar and both were significantly different from pretransfusion PCV, it is difficult from their study design to conclude that haematocrit equilibration process had been completed at 6 hours since they did not check PCV levels beyond 6 hours. Similarly, Adedoyin et al. [4] reported that 1-hour and 7-hour posttransfusion haemoglobin differed only by 0.2 g/dL but there were no estimated PCV levels after 7 hours. Further changes in PCV could have occurred beyond 7 hours after transfusion. Our study showed a posttransfusion haemoglobin difference of 0.4 g/dL between 1 hour and 12 hours, increasing to 0.9 g/dL by 24 hours.

Saugel et al. [11] reported a significant immediate and 2-hour increase in haematocrit among patients in ICU who received red blood cell transfusion. PCV check was, however, not repeated beyond 2 hours and the study, which was conducted among adult patients, was not designed to measure the duration of posttransfusion haemoglobin equilibration. Their strong support for immediate posttransfusion haemoglobin check may therefore not be entirely justified. This was however not the case with the findings of Elizalde et al. [12] who did posttransfusion PCV on 32 adults with normovolemic anaemia following bleeding episodes. Posttransfusion samples were taken at 15 min, 30 min, 60 min, 120 min, and 24 hours for PCV and no difference was found in the values, suggesting rapid haematocrit equilibration. Our study was carried out on neonates whose haemodynamic responses to transfusion may differ from those of adults.

While the differences in the duration of posttransfusion haematocrit equilibration process between our study and previous reports may not be easily explained, it can be speculated that the difference in the age group of patients in the various studies may be partly responsible. For instance Wielson et al. studied adults in the medical ward while Davies et al. retrospectively analyzed patients aged 1 day to 17 years and Saugel et al. analyzed only neonates. Furthermore, the duration of blood transfusion and the use of diuretics before or after transfusion may affect the haematocrit equilibration process. A large multicenter age-stratified study, specifically designed to analyze post-RBC transfusion haematocrit equilibration, would be required to resolve these differences.

### 4.1. Limitation to Study

We did not check the PCV in the interval between 6 and 12 hours after transfusion. The equilibration process could therefore have occurred at any point within this period. Doing that for this study would have considerably increased the number of times each baby was pricked for capillary blood sampling. We believe that this can be addressed in further studies specifically designed to check posttransfusion PCV values between 6 hours and 12 hours after red blood cell transfusion. A pilot study to determine the natural coefficient of variation for capillary PCV would have been quite helpful.

## 5. Conclusion and Recommendation

Our study has shown that, in stable nonhaemorrhaging and nonhaemolysing neonates, haemoglobin equilibration that probably begins during the period of transfusion ends by 12 hours after transfusion. Although the limited sample size and the seemingly marginal differences in the values of PCV between 1 hour and 12 hours after transfusion may limit the clinical application of our findings, we suggest that posttransfusion PCV be checked by 12 hours and recommend further studies involving larger sample sizes for further validation.

## Figures and Tables

**Table 1:** Demographic characteristics of subjects and cause of anaemia.

Gestational age distribution	*n* = 35 (%)
(i) <28 weeks	10 (28.6)
(ii) 29–33 weeks	4 (11.4)
(iii) 34–36 weeks	3 (8.6)
(iv) 37 weeks and above	18 (51.4)

Postnatal age at time of transfusion	*n* = 38

(i) 1–7 days	13 (34.2)
(ii) 8–14 days	5 (13.2)
(iii) 15–21 days	4 (10.5)
(iv) 22–28 days	4 (10.5)
(v) 29 days and above	12 (31.6)

Aetiology of anaemia	*n* = 40

(i) Anaemia of prematurity	16 (40)
(ii) Haemolysis	7 (17.5)
(iii) Postoperative	6 (15.0)
(iv) Birth trauma	1 (2.5)
(v) Others (not specified)	10 (25.0)

**Table 2:** Mean (SD) packed cell volume (PCV) at 1 hr, 6 hrs, 12 hrs, 24 hrs, and 48 hrs after transfusion.

	*P* value
PCV1 (48.1 ± 5.5%) versus	
PCV6 (47.8 ± 5.6%)	0.516
PCV12 (46.8 ± 5.9%)	0.081
PCV24 (45.9 ± 5.8%)	0.009
PCV48 (45.4 ± 6.2%)	0.005

PCV6 (47.8 ± 5.6%) versus	
PCV12 (46.8 ± 5.6%)	0.044
PCV24 (45.9 ± 5.8%)	0.010
PCV48 (45.4 ± 6.2%)	0.003

PCV12 (46.8 ± 5.6%) versus	
PCV24 (45.9 ± 5.8%)	0.237
PCV48 (45.4 ± 6.2%)	0.063

PCV24 (45.9 ± 5.8%) versus	
PCV48 (45.4 ± 6.2%)	0.403

**Table 3:** Mean target PCV versus mean observed posttransfusion PCV.

Posttransfusion time (hours)	Mean (SD) target-mean observed PCV (SD)	95% confidence interval	*t*	df	Significant level (2-tailed)
1 hour	−2.44 (6.68)	−4.88 to −0.029	−2.06	31	0.048
6 hours	−2.38 (6.46)	−4.70 to −0.046	−2.08	31	0.046
12 hours	−1.45 (5.66)	−3.49 to 0.59	−1.46	31	0.157
24 hours	−0.64 (5.25)	−2.53 to 1.25	−0.69	31	0.495
48 hours	−0.38 (5.14)	−2.23 to 1.48	−0.41	31	0.683
